# Population-based study of pharmacogenetics and pharmacokinetics in Southern African patients with multidrug-resistant tuberculosis (PoPG): a protocol for the Namibian cohort

**DOI:** 10.1136/bmjopen-2025-109764

**Published:** 2026-03-03

**Authors:** Lorraine Boois, Hilya Ekandjo, Olga Shavuka, Emmanuel Nepolo, Carene Anne Ndong Sima, Carola Oelofse, Caitlin Uren, Desiree C Petersen, Marlo Möller, Marie Wijk, Tracy Kellermann, Eric Decloedt, Helen McIlleron, Paolo Denti, Mareli Misha Claassens

**Affiliations:** 1 Faculty of Health Sciences and Veterinary Medicine, University of Namibia, Windhoek, Namibia; 2 Department of Biomedical Sciences, Stellenbosch University Faculty of Medicine and Health Sciences, Cape Town, South Africa; 3 South African Medical Research Council Centre for Tuberculosis Research, Stellenbosch University, Stellenbosch, South Africa; 4 Centre of Bioinformatics and Computational Biology, Stellenbosch University, Stellenbosch, South Africa; 5 Division of Clinical Pharmacology, University of Cape Town, Rondebosch, South Africa; 6 Division of Clinical Pharmacology, Stellenbosch University, Stellenbosch, South Africa; 7 Desmond Tutu TB Centre, Department of Pediatrics and Child Health, Stellenbosch University, Tygerberg, South Africa

**Keywords:** Africa South of the Sahara, Pharmacology, Genetics, Tuberculosis

## Abstract

**Abstract:**

**Background:**

Multidrug-resistant tuberculosis (MDR-TB) is an urgent public health challenge in Namibia, with profound socioeconomic consequences. The high burden of both tuberculosis and HIV complicates treatment and underscores the need for optimised drug therapies. Precision medicine, which leverages patient-specific genetic and molecular information, offers promise for improving MDR-TB outcomes. However, its effective application relies on population-specific data, particularly understanding how individuals metabolise tuberculosis drugs and how genetic diversity drives variability in treatment response. Currently, no pharmacokinetic (PK) or pharmacogenetic (PG) data on TB treatment exist for Namibian populations. This gap is particularly concerning, given the country’s genetic diversity, environmental factors and comorbidities that may uniquely influence drug metabolism. This study aims to generate PK and PG data to inform dose optimisation and support personalised treatment strategies for MDR-TB in Namibia. The findings will contribute to improved patient care and inform health system strengthening based on locally relevant evidence.

**Methods:**

This cross-sectional study will consist of 100 Namibian participants with matched human DNA and PK data of MDR-TB cases receiving isoniazid, clofazimine, bedaquiline and the fluoroquinolones (levofloxacin or moxifloxacin). PK sampling will be divided as follows: 30 individuals will undergo intensive PK sampling, while the remaining (n=70) will undergo sparse PK sampling. DNA will be extracted at Stellenbosch University (SU), and samples will be genotyped using the H3Africa microarray. Sequences will be aligned to the human reference genome, hg38 (GRCh38p13), using the freely available Burrows-Wheeler Aligner. A subset of the samples (n=20–30) will undergo whole genome sequencing (WGS) to verify imputation results and identify novel genetic variants potentially affecting PK in this population.

**Data analysis:**

Quality control and variant call format file generation will be performed using the Genome Analysis Toolkit best practices (V.3.5). Intensive and sparse PK data will be pooled for the development of a population PK (popPK) model using a non-linear mixed-effects modelling approach. The popPK model will characterise the relationship between TB drug dose and exposure, including quantifying covariates, including genetic variation, explaining PK variability, providing a foundation for dose optimisation and personalised treatment strategies.

**Ethics and dissemination:**

Ethics approval was obtained from the University of Namibia Human Research Ethics Committee for Health (Ref. SOM18/2024), the Ministry of Health and Social Services (Ref. 22/4/2/3), the SU Health Research Ethics Committee (Ref. N21/11/136) and the University of Cape Town Human Research Ethics Committee (Ref. 500/2022).

**STRENGTHS AND LIMITATIONS OF THIS STUDY**

- Utilises a mixed pharmacokinetic (PK) sampling strategy (intensive and sparse) to optimise data collection in a resource-limited setting.
- Incorporates genetic diversity from multiple Namibian ethnic groups into pharmacogenetic (PG)/PK analyses.
- Integrates PG data with clinical covariates for population PK modelling.
- Applies established imputation and sequencing pipelines to identify genetic variants influencing drug metabolism.
- Harmonises PK and genomic data with regional cohorts to enhance statistical power.

## Introduction

 Tuberculosis (TB) is the leading cause of death due to a single infectious agent, *Mycobacterium tuberculosis*. In 2023, approximately 8.2 million people were diagnosed with TB worldwide, resulting in 1.25 million deaths.^1 2^ Countries such as Namibia, South Africa, Zambia and Uganda are at the epicentre of a dual TB and HIV coepidemic. The emergence and spread of drug-resistant TB, especially multidrug-resistant TB (MDR-TB), which is resistant to both isoniazid (INH) and rifampicin (RIF), has significantly complicated TB control in high-burden countries.^1^ The number of MDR-TB cases has surged in recent years, yet effective management remains limited by delayed diagnosis, unclear resistance patterns, rigid treatment protocols and frequent adverse drug reactions (ADRs).^2^ In Namibia, MDR-TB accounts for an estimated 4.5% among new cases and 7.9% among previously treated patients, highlighting the urgent need for innovative approaches to TB control.^3^ One of the most critical interventions in TB control is chemotherapy, with the WHO recommending a standardised regimen of INH, RIF, ethambutol (EMB) and pyrazinamide.^1 4 5^ However, treatment outcomes remain highly variable, especially in resource-constrained settings, where success rates can fall below 70%—for instance, as low as 53% in parts of Nigeria.^6^ This variability is partly driven by individual differences in pharmacokinetics (PK) and pharmacogenetics (PG), which influence TB drug absorption, distribution, metabolism and excretion (ADME).^7^,^11^ Understanding these differences is critical for developing more effective and personalised treatment strategies, particularly in high-burden settings like Namibia.

Genetic variation in metabolism-related genes such as *NAT2* significantly impacts INH clearance. Slow acetylators (eg, *NAT2* *5 or *6) are at increased risk of drug-induced liver injury, while fast acetylators (eg, *NAT2* *4/*4) may not achieve therapeutic levels.^12^,^15^ When *NAT2* slow acetylator status is combined with *CYP2E1* fast metaboliser genotypes, the accumulation of toxic metabolites may increase, raising the risk of antituberculosis drug-induced liver injury (ATDILI).^15 16^ Variants in *SLCO1B1*, such as c.463C>A, have been linked to altered RIF exposure in African populations, while SLCO1B1 *15 has been associated with hepatotoxicity in Asian cohorts.^12 15 16^ Despite these insights, >95% of PK/PG studies to date are based on European and Asian populations, with African populations severely under-represented (~1%–2%).^17^,^19^ This gap raises concerns about extrapolating non-African findings to African settings, especially given the unique genetic structure and haplotype diversity of African populations.^11^,^1320 21^ Detecting novel variants is thus more likely in African cohorts (table 1).^7^,^9^ Namibia, in particular, offers a unique setting for PG/PK research due to its rich ethnic and genetic diversity. Study participants include Oshiwambo-, Otjiherero- and Khoekhoegowab-speaking groups, as well as San individuals, among others. The Khoe-San population, recognised as one of the most genetically diverse worldwide, may harbour novel, population-specific variants relevant to TB treatment outcomes.^22 23^ Investigating these understudied populations offers the potential to identify genetic determinants of interindividual variability and inform the development of more effective, locally tailored TB treatment strategies.^7 9 10 23^

**Table 1:** Significant (p<0.05) variant annotations assigned to INH, RIF, PZA and EMB, according to TBGxforAfrica Application^39^

Gene	Level of evidence	SNP	Impact/phenotype (drug)	No of studies	Significance range (p)	Populations	Reference
*AADAC*		rs1803155	Reduced exposure (RIF)	1	0.03	African	^40^
*SLCO1B1*	3	rs11045819	Altered clearance (RIF)	1	0.0009	African	^41^
*SLCO1B1*	3	SLCO1B1*15 (rs4149056)	ATDILI (RIF)	1	0.007	Asian	^42^
*NOS*	3	rs11080344	ATDILI (INH, RIF)	1	0.036	East Asian	^43^
*NR1I2*	3	rs2472677	Peripheral nervous system (INH, RIF)	1	0.018	African	^44^
*NR1I2*	3	rs2472677	Death (INH, RIF)	1	0.007	African	^44^
*RIPOR2*	3	rs10946739	ATDILI (RIF)	1	0.0000041	African	^45^
*RIPOR2*	3	rs10946737	ATDILI (RIF)	1	0.00000044	African	^45^
*CUX2*	3	rs7958375	ATDILI (INH, RIF, EMB, PZN)	1	0.000012	Sub-Saharan African	^45^
*TNFa*	3	rs1800629	ATDILI (INH, RIF, EMB, PZN)	1	0.034	East Asian	^46^
*AGBL4*	3	rs393994	ATDILI (INH, RIF, EMB, PZN)	1	0.0000076	Sub-Saharan African	^45^
*AGBL4*	3	rs320003	ATDILI (INH, RIF, EMB, PZN)	1	0.0000083	Sub-Saharan African	^45^
*AGBL4*	3	rs319952	ATDILI (INH, RIF, EMB, PZN)	1	0.0000085	Sub-Saharan African	^45^
*CYP3A5*	2	rs776746	Reduced BDQ exposure	1	0.0017	Sub-Saharan African	^12^
*CYP2B6*	2	*C*YP2B6*6 (rs3745274)	ATDILI	2	0.02	Asian	^47^
*SLCO1B1*	3	rs4149056	Altered RIF clearance	1	<0.001	Asians	^42^
*SLCO1B1*	3	rs4149032	Reduced RIF exposure	1	Not reported	Sub-Saharan African	^48^
*NAT2*	(1–3) varies	NAT2*14 (rs1801279), NAT2*5 (rs1801280), NAT2*6 (1799930), NAT2*7 (rs1799931)	Slow acetylator status (INH toxicity)	20+	0.0001–0.05	African, Asian European	^49^
*CYP2E1*	(2–3) varies	rs2031920	Modifies INH toxic metabolite formation	10+	0.001–0.05	Asian	^50^
*GSTM1*	2–3	Gene deletion (null genotype)	Increased risk of ATDILI	10+	<0.05	Asian	^51 52^
*GSTT1*	2–3	Gene deletion (null genotype)	Increased risk of ATDILI	10+	N/A	Asian	^51^
*CYP2C9*	2	rs9332096	Drug-induced maculopapular eruption (INH, RIF)	5+	0.022	Asian	^53^
*CYP2C19*	2	rs4986893	Drug-induced maculopapular eruption (INH, RIF)	5+	0.042	Asian	^53^
–		–	**No well-characterised PG markers currently identified available (CFZ, delamanid (DLM), LFX, LZD)**			–	–

Studies including African populations are indicated in green. While extensive PG associations have been reported for first-line drugs used in DS-TB, evidence for second-line drugs used in MDR-TB remains sparse. Few studies have examined genetic variants affecting metabolism of newer drugs like BDQ. Notably, variants in CYP3A5 and CYP2B6 have been linked to altered BDQ metabolism, yet broader PG data for MDR-TB regimens are lacking—particularly in African populations.

ATDILI, antituberculosis drug-induced liver injury; BDQ, bedaquiline; CFZ, clofazimine; DS-TB, drug-sensitive tuberculosis; EMB, ethambutol; INH, isoniazid; LFX, levofloxacin; LZD, linezolid; MDR-TB, multidrug-resistant tuberculosis; PG, pharmacogenetics; PharmGKB, Pharmacogenomics Knowledge Base; PZA, pyrazinamide; PZN, pyrazinamide; RIF, rifampicin; SNP, single nucleotide polymorphism.

To address this gap, we propose the establishment of a Namibian cohort with matched DNA and PK data, in collaboration with the University of Namibia (UNAM), Stellenbosch University (SU) and the University of Cape Town (UCT). This study is part of a larger initiative, the Population-Based Study of Pharmacogenetics and Pharmacokinetics in Southern African Patients with Tuberculosis (PoPG), encompassing Namibia, South Africa, Zambia and Uganda. Our objectives are to (1) generate a genetic dataset (n=100) with deep phenotyping, including PK and adverse event (AE) data and (2) identify novel PG variants through advanced computational methods and whole genome sequencing (WGS) of Namibian populations. This effort aims to inform the development of personalised MDR-TB therapies, strengthen healthcare systems and support locally relevant regulatory and clinical decision-making, particularly in high-burden, ethnically diverse and resource-limited settings.^20^

## Methods and analysis

### Patient and public involvement

The Namibian cohort of PoPG will be nested in an existing ongoing project (Hotspots, households and hospitals: enhanced MDR-TB case finding in Namibia (H3TB)) funded by British Medical Research Council (MRC)/Department for International Development (DFID) and European and Developing Countries Clinical Trials Partnership (EDCTP).^24^ Notably, H3TB has collaborated directly with the National TB and Leprosy Programme (Dr Nunurai Ruswa) and with the national reference laboratory, the National Institute of Pathology (NIP) (Ms Queen Mokomele). The protocol of PoPG was developed in collaboration with SU and UCT. Moreover, H3TB and PoPG are coordinated by the Group for Research in Infectious Diseases (GRID) at UNAM. GRID is in the process of establishing a community advisory board. Furthermore, the research ethics committee of UNAM will incorporate community members from 2025.

### Design

This is an observational study. As this study is nested in an existing ongoing study, the protocol will be adopted with minor adjustments.^24^ While formal sample size calculations were not performed due to the exploratory nature and the inclusion of multiple drugs, the selected sample size and sampling schedule are expected to be adequate based on previous studies.^8 25^ Importantly, this study contributes Namibian data to a broader regional effort—the PoPG. The estimated total sample size of >1500 participants corresponds to the target of the regional PoPG study, encompassing cohorts from Namibia, South Africa, Zambia, Botswana and Uganda. This estimate was derived from the cumulative total of participants across all contributing PK, PG and population genetic studies available from collaborating sites. These datasets include banked samples from existing cohorts with matched PK and AE data, representing the largest consolidated African TB PG dataset currently available. Population PK studies have demonstrated adequate power to detect meaningful PG/PK associations even in cohorts of 50–100 individuals. A sample size exceeding 1500, therefore, ensures robust statistical power across multiple drugs while accommodating intercohort variability in drug exposure and ancestry composition.

For the Namibian cohort—the only site with newly collected samples—100 participants will contribute PG/PK data. A subset of approximately 20–30 participants (~2% of the cohort) will undergo WGS at the South African Medical Research Council (SAMRC) Genomics Centre to verify imputation results and assess imputation accuracy across ADME genes. The subset for WGS will be selected to maximise genetic diversity and represent the breadth of Southern African ancestry, particularly including individuals with KhoeSan, Damara, Ovambo, Tswana and Herero ancestry. Selection will be stratified to reflect major ethnic and genetic groups represented in the cohort, ensuring maximal capture of population diversity and allele frequency variation, and will prioritise ethnic and geographic representation, data completeness and DNA quality, as well as distinct PK profiles to capture phenotypic extremes. By pooling existing PK data and integrating PG analyses, this initiative aims to improve the understanding of drug metabolism variability across Southern African populations.

### Setting

The study discussed here will be conducted in Namibia in three regions, namely Khomas, Ohangwena and Otjozondjupa. Recruitment will take place between the period August 2025 and December 2026. Sample processing capacity differs not by region but by the infrastructure available at specific sites. In Khomas, samples collected from TB patients at Katutura Intermediate Hospital can be transported within minutes to the nearby UNAM Molecular Diagnostics Laboratory, which is equipped with a −80℃ freezer. This allows same-day processing and storage, reducing the risk of degradation—particularly important for drugs such as INH, which has limited stability in whole blood and plasma.^15^

In contrast, sites such as Ohangwena and Otjozondjupa do not have local access to ultra-low temperature storage. Samples from these regions will be centrifuged within 1 hour of collection using a portable centrifuge and temporarily stored in a −20℃ portable freezer powered by a mobile power station. Daily transport will be arranged through third-party services capable of maintaining a cold chain using dry ice, ensuring delivery to a central laboratory within 48 hours for final storage at −80℃. While this setup safeguards data quality, the absence of immediate ultra-cold storage in remote areas may compromise the accuracy of INH concentration measurements. Procuring a portable −80℃ freezer for field use would help maintain sample integrity and improve PK data reliability.

### Participants

For our Namibian study, we anticipate recruiting 100 participants. As this study is nested within H3TB, the population identification process will be replicated.^24^ Potential participants with a positive GeneXpert/RIF as recorded by the NIP, indicating rifampicin resistance, and a positive *tuberculosis* culture will be included from three regions (Khomas, Otjozondjupa and Ohangwena). To ensure accurate and reliable PK analysis, study participants must have been on standard MDR-TB chemotherapy for a minimum of 4–8 weeks following treatment initiation. Additionally, participants will be required to have received at least one of the target TB drugs—INH, clofazimine (CFZ), bedaquiline (BDQ) or the fluoroquinolones levofloxacin (LFX) or moxifloxacin (MOX)—for this duration. Participants will be selected consecutively from the H3TB parent cohort as they meet eligibility criteria, until the target sample size is reached.

### Outline

**Figure 1:**
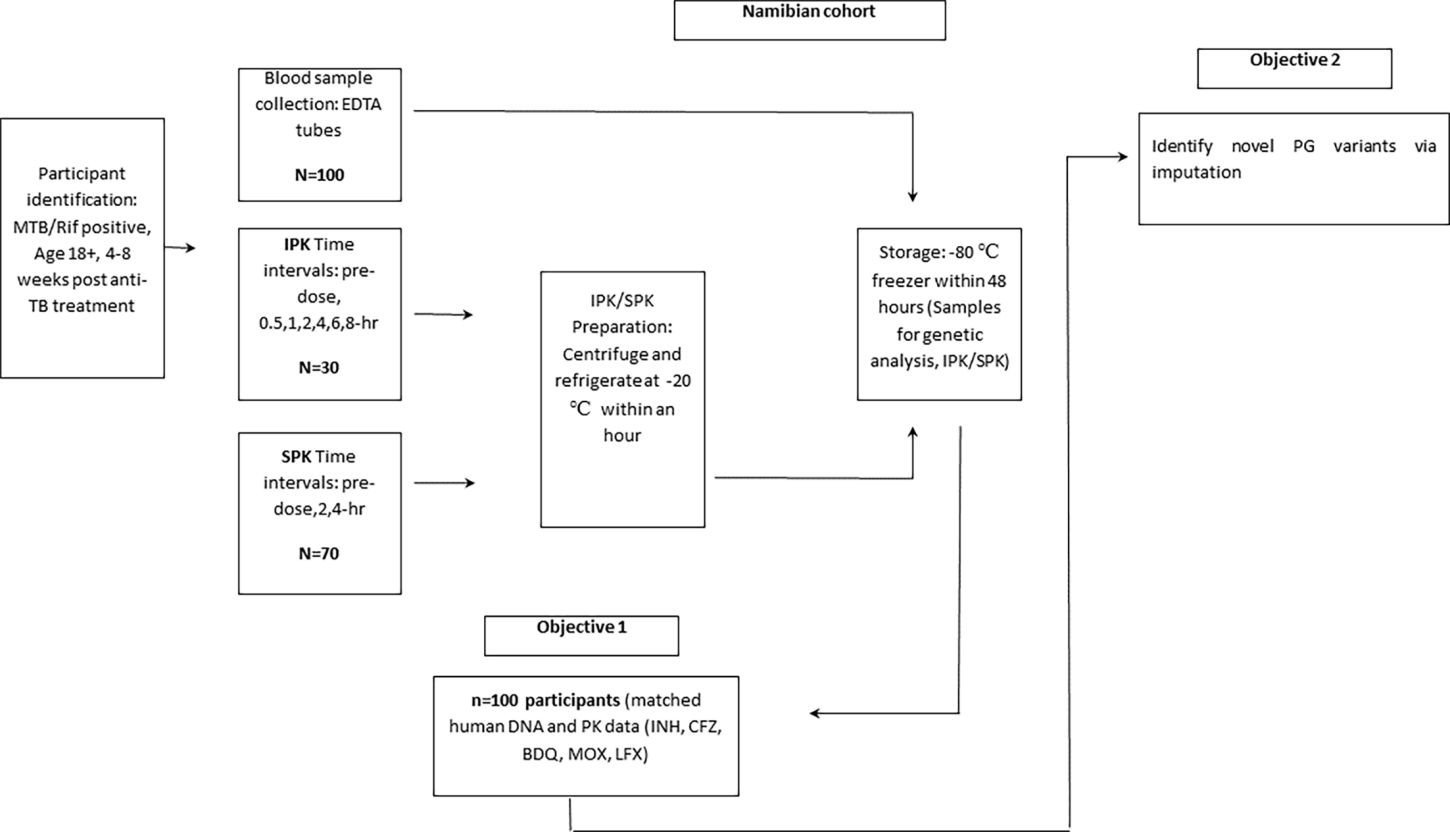
Study outline. BDQ, bedaquiline; CFZ, clofazimine; INH, isoniazid; IPK, intensive pharmacokinetics; LFX, levofloxacin; MOX, moxifloxacin; MTB, Mycobacterium tuberculosis; PG, pharmacogenetics; PK, pharmacokinetics; Rif, rifampicin; SPK, sparse pharmacokinetics; TB, tuberculosis.

Written informed consent will be obtained by trained staff from individuals meeting the eligibility criteria mentioned above. In addition, participants are required to be ≥18 years of age at the time of recruitment. Women will be asked about pregnancy status and will be excluded if pregnant. Blood samples will be taken at different time intervals throughout the day. Counselling and rapid HIV tests will be offered to participants with unknown HIV status. This will be conducted to recognise the possible impact on the effectiveness of drug-to-drug interactions on TB drugs for patients on TB treatment and antiretroviral treatment (ART). In certain cases, multiple visits will be made. Compensation of N$150 (~USD10) per session will be provided. A session for this study will be defined as the completion of the collection of all blood samples required for PG/PK data generation and analysis from potential participants (see figure 1).

### Data access and management

Tablets will be used to enrol participants as well as to capture identifying information in a RedCap database. Barcodes will be used to link enrolment and blood samples. As there is an existing Memorandum of Understanding between UNAM and SU, the acquired Namibian human biological samples will be analysed and stored in a biorepository based at SU. Currently, there is no biorepository in Namibia; however, this project will be fundamental in building this capacity. In addition, sequencing analysis results not obtained at UNAM, namely WGS, genotyped data and PK data, will be transferred securely to the RedCap Server based at SU, as UNAM is currently in the process of building the capacity to perform the latter in the future, in a combined database. Clinical data and routine laboratory data (Xpert MTB/Rif, culture, drug sensitive test) will be obtained from participants’ questionnaires and ‘eTB manager’, the national MDR-TB database, and ‘Meditech’, a software platform used by NIP for record tracking of results, and stored in a database at the UNAM server.

Data will be analysed with STATA and R software. For the final analysis of the dataset, the sequencing data obtained at SU, the WGS data from SAMRC Genomics Platform, PK/PG data from UCT and the clinical data obtained at UNAM will be merged in a combined database. Data will be stored in an anonymised RedCap database that will be available to project leaders subject to regular monitoring. All data will be deidentified before submission to the shared databases, and identifying details will not be directly linked to the dataset. Moreover, this information will be kept separate with limited access on the UNAM server. Data will be deidentified using the Safer Harbour Method: (https://www.hhs.gov/hipaa/forprofessionals/privacy/specialtopics/deidentification/index.html#standard). Researchers will be required to sign confidentiality agreements. In addition, study staff will undergo Good Clinical Practice training. It will be compulsory for researchers to familiarise themselves with the data management plan standard operating procedure prior to the commencement of the study. Participant confidentiality violations will be acted on accordingly.

### PK plasma sample collection, processing and analysis

Blood collection for PK analysis is preferable 4–8 weeks post-TB treatment initiation, as this time frame allows for the reduction of interindividual variability due to early disease response and treatment adjustment. Most of the drugs under study, with the exception of CFZ and BDQ, will have reached steady-state plasma concentrations, which is critical for meaningful PK assessment. By limiting sampling to this postinitiation window, we reduce variability due to drug accumulation (with the exception of CFZ and BDQ), which is not easily accounted for using non-compartmental analysis. It also improves the reliability of genotype to phenotype correlations, as the effects of PG variants are more clearly observed once initial metabolic fluctuations stabilise.

The regional PoPG dataset includes first- and second-line anti-TB drugs—INH, CFZ, BDQ and fluoroquinolones (LFX/MOX). The planned distribution of participants by drug is approximately INH (n=1000), CFZ (n=400), BDQ (n=300) and fluoroquinolones (n=300), with overlap for participants receiving combination therapy. Data collected include PK, PG and AE information. Safety events will be prospectively captured using standardised AE reporting forms aligned with WHO Toxicity Grading Scales and recorded in REDCap for linkage with PK/PG data in exploratory analyses. Expected safety events include hepatotoxicity, QT interval prolongation, skin and gastrointestinal reactions, neurological AEs and general drug intolerance. Only AEs with frequencies ≥5% will be analysed for PG associations, acknowledging that exploratory analyses may have limited power for rare events.

Participants will be identified progressively during the month of diagnosis. Both hospitalised and discharged outpatients will be eligible. If discharged, field teams will trace participants to their households for sample collection. In areas with multiple eligible outpatients, participants will be contacted (by phone or in person) a day before and transported to a central location for sampling, such as the UNAM Hage Geingob Campus in Khomas. Each participant will be asked to record the time and dose of their most recent TB drug intake prior to the PK sampling visit, while the dose on the day of PK will be administered by the study staff. Blood samples will be collected at specified intervals for either intensive (predose, 0.5 hour, 1 hour, 2 hours, 4 hours, 6 hours and 8 hours) or sparse (predose, 2 hours, 3 hours and 4 hours) PK sampling.

Blood samples will be centrifuged at 3000 rpm for 10 min and plasma aliquoted into prelabelled polypropylene microcentrifuge tubes (1.5 or 2 mL). In Khomas, plasma will be transferred to a −80℃ freezer within 1 hour. In Otjozondjupa and Ohangwena, samples will first be stored in a portable −20℃ freezer and transported on dry ice to the nearest −80℃ freezer facility through third-party cold-chain logistics services. All sample processing times, centrifugation and freezing, will be documented. Drug concentrations for INH, CFZ, BDQ and LFX/MOX will be quantified using liquid chromatography mass spectrometry at the Division of Clinical Pharmacology, UCT.

All contributing cohorts have ethics-approved, biobanked DNA and plasma samples, with custodians listed as coinvestigators on the proposal. These samples are stored in the Hamilton BiOS automated biorepository at SU and equivalent certified facilities at partner institutions. To ensure sample integrity, all transfers will be conducted under strict cold-chain conditions, following a detailed chain-of-custody protocol.

We acknowledge heterogeneity in treatment regimens and drug exposure across studies. To mitigate this, all datasets will undergo harmonisation within a unified RedCap database to capture PK parameters, AEs, drug regimens and relevant covariates, such as HIV status, comedications and ART exposure. Population PK modelling (using NONlinear Mixed Effects Modeling (NONMEM)) will be applied to adjust for study-level and drug-specific variability, enabling identification of genetic effects independent of confounding clinical factors. Study-specific characteristics, including assay methods and dosing differences, will be modelled as random effects to minimise bias and prevent spurious associations. Sensitivity analyses will further evaluate the influence of between-site variability on PK parameters.

#### PG/PK data modelling

The drug concentration time data will be analysed using population PK (popPK) modelling, a non-linear mixed effects approach widely applied in studies with sparse or flexible sampling designs (44). This method is particularly appropriate in this study, where certain drugs, especially those with long half-lives such as BDQ and CFZ, have limited sampling points that prevent the development of new PK models. In such cases, existing models will be adapted and updated using data from prior studies and the PoPG study. While the Khoe San population is genetically distinct and under-represented in PG studies of MDR TB treatment, the decision to use popPK is not based on this novelty alone. PopPK enables the simultaneous assessment of PG variants such as NAT2 polymorphisms affecting INH clearance and clinical covariates, including age, antiretroviral cotreatment, renal function and weight, within a single integrated model. This approach provides a more comprehensive understanding of variability in clearance and bioavailability than analysing these factors separately.

Model development will be developed using NONMEM,^25^ supported by Pirana, Perl speaks NONMEM and Xpose4.^14^ Where appropriate, previously developed models from similar populations will be used as a foundation.^19 25 26^ Structural models (eg, one or two compartments with first-order absorption and elimination) will be tested, followed by inclusion of between-subject and between-occasion variability using log-normal distributions.^27^ Allometric scaling will adjust for body size, using either total body weight or fat-free mass, with fixed exponents of 0.75 for clearance and 1 for volume.^28^ Covariates such as age, antiretroviral cotreatment and renal function will be considered for inclusion in the population PK model based on their biological relevance. Their impact will be assessed visually using diagnostic plots (eg, predictive checks) and statistically by testing whether adding each covariate leads to a significant decrease in the objective function value (OFV). The OFV is assumed to follow a χ² distribution, allowing determination of statistical significance. Covariates will be selected through a stepwise process, where only those that cause meaningful improvements in model fit, reflected by reductions in OFV, are retained.

Genetic effects will be incorporated into the PK model in two stages. Initially, known PG markers (eg, *NAT2* polymorphisms affecting INH clearance) will be tested as covariates. Subsequently, unexplained between-subject variability in key parameters such as clearance and bioavailability will be explored by regressing empirical Bayes estimates against a set of preselected candidate SNPs. These candidate SNPs will be chosen based on prior evidence from the literature and biological plausibility to limit the number of tests and reduce false positives. Promising associations identified through this screening will then be formally incorporated into the model as covariates to quantify their influence on drug PK. This approach balances leveraging known functional variants with discovering novel genetic contributors while maintaining statistical rigour.

#### PG algorithm for effective drug dosage

To optimise initial TB drug dosing, a PG algorithm will be developed based on significant associations identified between PK parameters, genetic variants and clinical outcomes. The aim of the algorithm is to predict drug initiation doses that achieve therapeutic drug exposure efficiently, minimising time to target concentrations. An ancestry-aware genomic framework will be employed to disentangle the relative contributions of clinical, PK and genetic factors to interindividual variability in drug response. This will involve the use of stratified linkage disequilibrium (LD) score regression models to estimate the heritability of drug response attributable to genetic ancestry, as well as PK and clinical parameters.

The proposed PG algorithm will integrate clinical, PK and genetic data to predict individualised dosing that achieves target drug exposure. The algorithm will be developed for anti-TB drugs included in the study—INH, CFZ, BDQ and fluoroquinolones (LFX/MOX)—initially focusing on INH and BDQ, where PG markers are known or suspected to influence clearance. Development will proceed through four stages: (1) discovery cohort (~70% of data): population PK and mixed-effects models (NONMEM) will identify significant genetic predictors of clearance and bioavailability; (2) validation cohort (~30% of data): model predictive performance will be tested and refined; (3) machine learning integration**:** Bayesian mixed-effects and stratified LD score regression models will estimate the proportion of dose–response variability explained by genetics, ancestry and clinical covariates and (4) verification**:** key variants influencing dose–response will be confirmed by Sanger sequencing and cross-compared with WGS-derived variants. Algorithm development will use a Bayesian mixed-effects modelling framework integrating clinical covariates, genetic variants and PK parameters to predict dosing that achieves therapeutic targets rapidly. Internal validation will be performed using cross-validation and simulation-based diagnostics within the Namibian dataset, while external validation will leverage data from other PoPG sites. The final outputs will include ancestry-informed dosing recommendations and a preliminary predictive algorithm to guide future clinical validation in Southern African cohorts.

Advanced probabilistic modelling techniques, including Bayesian inference, Dirichlet processes and Monte Carlo sampling, will be applied to account for uncertainty, model population structure and improve the robustness and accuracy of dose predictions. The final PG algorithm will integrate these components into a clinically interpretable tool to support personalised dosing strategies in TB treatment.

#### Power calculation

It is not possible to offer a meaningful sample size calculation, as the statistical power within our analyses varies completely between different drugs and depends on the prevalence of the genetic polymorphism of interest, on the size of its effect, etc. However, population PK has been shown to be quite powerful in identifying PG/PK effects already with cohorts of sample size of 50 to 200 patients.^29^,^33^ In addition, model-based approaches can be used to perform stochastic simulation and estimation to accurately calculate the power of the design and data included in the model. Herewith, being able to report with which confidence major effects (eg, >50% difference in confidence level) with a certain prevalence in population (eg, 20% of patients carrying the specific SNP) can be excluded.

### Laboratory methods for sequencing

#### DNA extraction

DNA will be extracted from blood samples using standard methods at SU, Cape Town, South Africa for further analysis.

#### Genotyping and imputation

H3Africa microarray genotyping will be performed commercially at the Centre for Proteomic and Genomics Research in Cape Town, South Africa. Raw genotyping data will undergo rigorous analysis-specific quality control checks in line with previously published pipelines specific to the array and genotyping platform used. Genotype imputation is a powerful tool for increasing statistical power in genetic analyses.^34^ Meta-analysis of multiple study datasets also requires a substantial overlap of SNPs for a successful analysis, which can be achieved by imputation. Quality of imputed datasets is largely dependent on the software used, as well as the reference populations chosen. It has previously been shown that the African Genome Resource is the best reference panel for imputation of missing genotypes in samples from admixed South African populations, implemented via the freely accessible Sanger Imputation Server 8. We will use this established pipeline together with previously collected WGS data to impute the genotyping data generated, as the Infinium H3Africa Consortium array v2 from Illumina will not allow direct genotyping of all relevant variants.

This approach will also allow us to identify novel genetic variants that may affect PK. As the number of applicable reference populations and individuals grows, imputation accuracy will improve for African and admixed populations, but it remains the gold standard to sequence a variant of interest to confirm that the imputed variant is present in the population prior to conducting further research. For this reason, PoPG will do WGS for a subset of participants (n=20–30) to confirm the imputation results. Following imputation, the genotypic data will be phased before undergoing global ancestry inference using ADMIXTURE and RfMix. Both of these tools have been shown to be highly accurate in African populations.^35^ Results will be collated in an anonymised allele frequency database (https://tbpgxforafrica.shinyapps.io/PGxForAfrica/).

#### Sequencing

The DNA of the previously mentioned subset of participants, n=20–30 (selected to encompass the genetic diversity of the PoPG dataset as a whole) will be whole genome sequenced at the SAMRC Genomics Centre using the MGISEQ-2000rs or the DNBSEQ-G to verify the imputation results.^36^

#### Generation and harmonisation of PK and AE data in RedCap database

**Figure 2:**
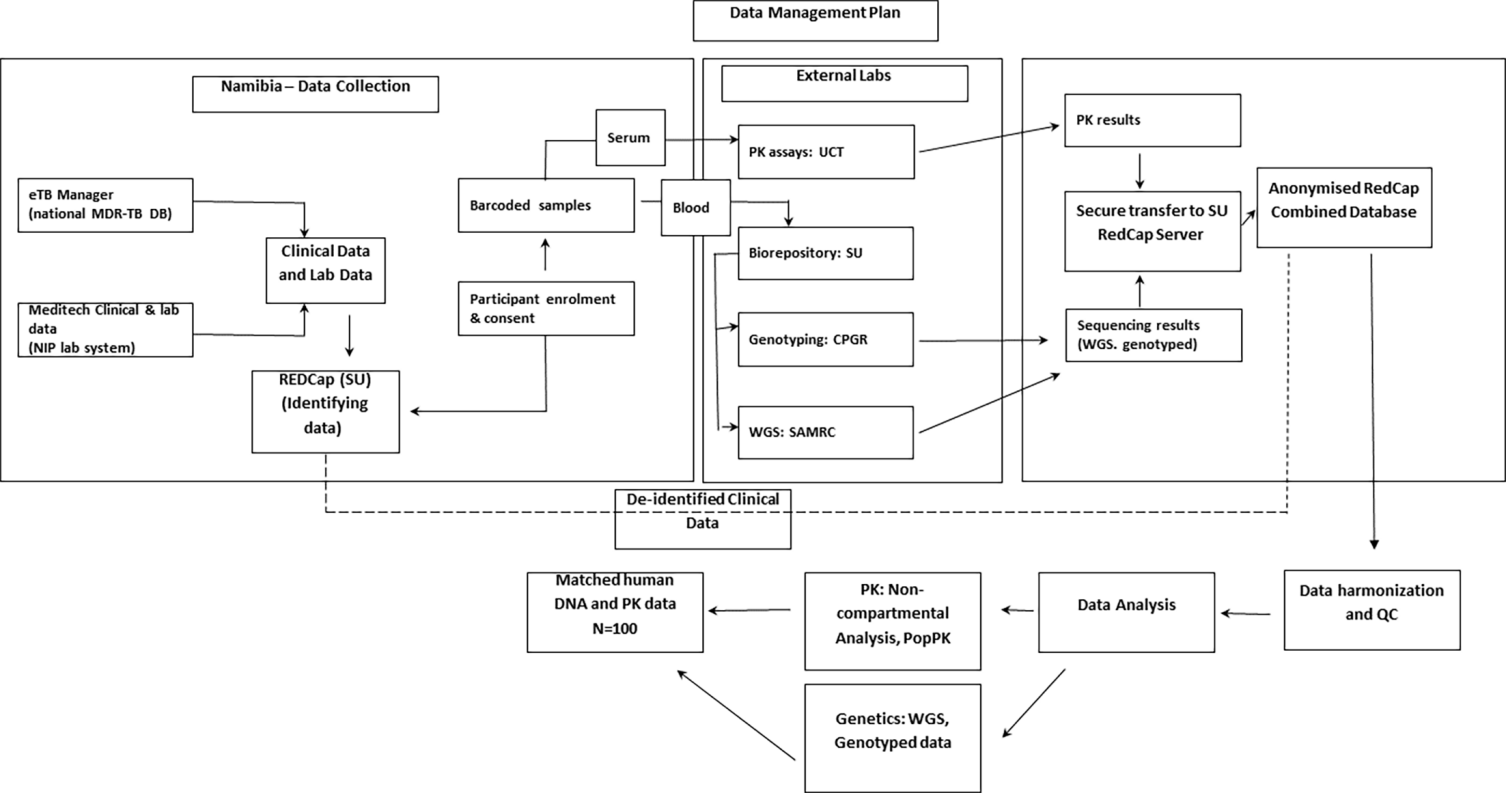
Schematic of data and sample flows, analysis sites, and the final merged anonymised dataset used for PK/PG analyses. Arrows indicate sample/data movement and processing steps. CPGR, Centre for Proteomic and Genomics Research; DB, database; eTB, electronic tuberculosis; MDR-TB, multidrug-resistant tuberculosis; PK, pharmacokinetics; PopPK, population pharmacokinetics; QC, quality control; SAMRC, South African Medical Research Council; SU, Stellenbosch University; UCT, University of Cape Town; WGS, whole genome sequencing.

Population PK models will be developed initially using the intensive PK data, followed by incorporation of the sparse PK data. For the final analysis, the combined database from our study, comprising genotyping data, WGS data, PK, AEs and clinical parameters, will be harmonised with data from other related studies within a REDCap database (see figure 2). This harmonisation process will include data merging and quality control steps. From the integrated dataset, TB drug-specific and/or ethnicity-specific cohorts will be formed. Any variations in treatment regimens, concomitant medications or relevant clinical information, such as HIV status, will be captured and adjusted for within the population PK models to ensure accurate interpretation.

#### Bioinformatics and statistical analysis

All bioinformatics analysis for WGS will be conducted using the computational equipment available at the Centre for High Performance Computing, based at the Council for Scientific and Industrial Research. Sequences will be aligned to the human reference genome, hg38 (GRCh38p13),^27^ using the freely available Burrows-Wheeler Aligner. Quality control and variant call format file generation will be performed using the Genome Analysis Toolkit best practices (V.3.5).^36^

### Ethics

Ethics approval was obtained from the University of Namibia Human Research Ethics Committee for Health (Ref. SOM18/2024), the Ministry of Health and Social Services (Ref. 22/4/2/3), the SU Health Research Ethics Committee (Ref. N21/11/136) and the UCT Human Research Ethics Committee (Ref. 500/2022). Written informed consent will be obtained from all participants prior to enrolment. Data obtained from the analysis will give critical insights into future TB treatment, and it will serve as a role model for similar studies in other high-incidence settings. The study results will be submitted to a peer-reviewed journal.

## Discussion

The rapid generation of GWAS and PG/PK data related to African populations is essential for optimising drug therapy, informing drug development, and ultimately enabling precision medicine.^9^ Despite their high genetic diversity and population-specific variants, African populations remain vastly under-represented in existing genetic datasets.^17^ This under-representation limits the global applicability of precision medicine and exacerbates health inequities.^20^ Large-scale genetic datasets are urgently needed to advance personalised medicine efforts and reduce the burden of ADRs and ineffective treatments—particularly in resource-limited healthcare systems.^9^

In the context of MDR-TB, where treatment regimens are complex, toxic and often prolonged, personalised medicine offers a powerful avenue to improve patient outcomes. By identifying genetic factors that influence drug metabolism and response, individualised treatment plans could enhance efficacy, reduce AEs and improve adherence—ultimately supporting better cure rates.^7^ However, routine genotyping for pharmacogenetically relevant variants remains uncommon across much of the African continent, and implementation of precision medicine remains in its early stages.

Given the resource constraints and lack of existing data in countries such as Namibia, generating context-specific GWAS and PG/PK data presents a unique challenge. This study aims to serve as a foundational step towards building local capacity for genomic research and integrating precision medicine into TB care in Namibia and other high-burden settings. The locally generated data will contribute to addressing current knowledge gaps and align with major continental initiatives, including Human Heredity and Health in Africa (H3Africa), the African Genome Variation Project, the African Pharmacogenetics Consortium, and the Deutscher Akademischer Austauschdienst (DAAD) In-Region Scholarship Programme in Molecular Biology and Human Genetics.^37^ Furthermore, the project will foster academic development through peer-reviewed publications, capacity-building and student mentorship, thereby contributing to the advancement of African-led genomic research.^38^ In doing so, it will not only advance African-led genomic research but also bring Namibia one step closer to realising the benefits of personalised medicine for MDR-TB and beyond.
