# Supplementary material for: Population-based study of pharmacogenetics and pharmacokinetics in Southern African patients with multidrug-resistant tuberculosis (PoPG): a protocol for the Namibian cohort
Source: BMJ Open. 2026 Mar 3;16(3):e109764. doi: 10.1136/bmjopen-2025-109764 (PMC12959021; doi:10.1136/bmjopen-2025-109764)
Supplement: Supplementary file 1 [file bmjopen-2025-109764.reviewer_comments.pdf]

PEER REVIEW HISTORY

BMJ Open publishes all reviews undertaken for accepted manuscripts. Reviewers are asked to complete a checklist review form and are provided with free text boxes to elaborate on their assessment. These free text comments are reproduced below.

ARTICLE DETAILS

Title (Provisional)

A population-based study of pharmacogenetics and pharmacokinetics in Southern African patients with multi drug resistant tuberculosis (PoPG): a protocol for the Namibian cohort

Authors

Boois, Lorraine; Ekandjo, Hilya; Shavuka, Olga; Nepolo, Emmanuel; Ndong Sima, Carene Anne; Oelofse, Carola; Uren, Caitlin; Petersen, Desiree C; Möller , Marlo; Wijk, Marie; Kellermann, Tracy; Decloedt, Eric; McIlleron, Helen; Denti, Paolo; Claassens, Mareli Misha

---

This article was not externally reviewed at BMJ Open. Protocols that have been independently assessed prior to submission to BMJ Open are usually fast-tracked to publication on the grounds that further substantial changes will not be possible. This independent assessment will usually be external, independent review for both a substantial grant award from a non-commercial or government funder and ethics approval.
